# Genetic insights into aging: Obesity’s impact on frailty risk uncovered

**DOI:** 10.1097/MD.0000000000046406

**Published:** 2026-01-02

**Authors:** Yongguang Wang, Jinlei Zhou, Tingxiao Zhao, Qi Wang

**Affiliations:** a Department of Orthopedics, Hangzhou Linping District Hospital of Integrated Traditional Chinese and Western Medicine, Hangzhou, Zhejiang Province, China; b Center for Plastic & Reconstructive Surgery, Department of Orthopedics, Zhejiang Provincial People’s Hospital (Affiliated People’s Hospital, Hangzhou Medical College), Hangzhou, Zhejiang Province, China.

**Keywords:** BMI, causal relationship, frailty, Mendelian randomization study, obesity, waist circumference

## Abstract

Frailty is an age-associated condition characterized by a state of vulnerability that compromises the quality of life and independence of older adults. To date, no studies have employed Mendelian randomization (MR) to investigate the causal relationship between obesity and frailty risk. Therefore, this study utilized a two-sample MR approach to elucidate the potential causal link between obesity and frailty. A two-sample MR analysis was conducted to assess the causal relationship between body mass index (BMI) or waist circumference and frailty. Independent genetic variants associated with obesity and frailty were selected as instrumental variables (IVs). MR analyses were primarily performed using inverse variance weighting, MR-Egger, weighted median, simple, and weighted models. In addition, the Mendelian Randomization Pleiotropy RESidual Sum and Outlier framework was applied to assess horizontal pleiotropy and to identify potential outlier variants. Through a rigorous and meticulous screening process, we identified 68 and 41 single nucleotide polymorphisms as IVs for BMI and waist circumference, respectively. Our analyses uncovered a significant positive causal association with frailty for both BMI (β = 0.1283, SE = 0.0255, *P* = 5.2589e−07) and waist circumference (β = 0.1340, SE = 0.0357, *P* = .0001). Cochran *Q*-test indicated the presence of heterogeneity among the IV estimates attributable to individual variant effects in both analyses (*Q* = 153.8575, *P* < 8.753493e−09; *Q* = 111.1552, *P* < 7.432416e−09). Besides, no significant pleiotropy was detected for the association of BMI, waist circumference and with frailty (intercept = 0.002609868, *P* = .2054007; intercept = 0.003445858; *P* = .7968586). The Mendelian Randomization Pleiotropy RESidual Sum and Outlier analysis identified no outliers for either exposure, and thus no single nucleotide polymorphisms were excluded. This study substantiates the significant association between obesity and an elevated risk of frailty, thereby offering a robust theoretical foundation for policymakers to institute more stringent weight management strategies.

**Key points**

- Genetic prediction of a causal relationship between obesity and frailty.
- Two-sample Mendelian randomization (MR) analysis showed a causal relationship between obesity and risk of frailty.

## 1. Introduction

In light of the burgeoning challenge of worldwide demographic senescence, there is an increasing pervasiveness of age-related pathologies.^[1]^ Frailty, recognized as a geriatric syndrome, embodies a complex interplay of multifarious pathophysiological processes, conferring heightened vulnerability to detrimental health outcomes upon encountering extrinsic or intrinsic pathogenic stimuli.^[2,3]^ Consequences intricately associated with frailty include functional impairment, falls, osteoporotic fractures, hospital admissions, and mortality.^[4]^ A prevalent methodology to gauge frailty involves calculating a Frailty Index predicated upon the ratio of accrued health deficits in individuals aged 3 years and above. Meta-analytical observations delineate a frailty prevalence of 26.8% within a sample of 56,407 geriatric participants with a mean age of 78.59 years, originating from Western countries.^[5]^

Adipose tissue is recognized to be profoundly interwoven with inflammatory and immune responses, as it actively exudes both pro-inflammatory and anti-inflammatory cytokines, endocrinologically active compounds, and chemotactic agents.^[6,7]^ Individuals manifesting overweight or obese conditions may harbor a heightened susceptibility to frailty, with excess body weight postulated as a probable precipitating factor for frailty onset.^[8,9]^ Overweight status, as measured by the body mass index (BMI) and waist circumference, corresponds to an aberrant accumulation of adipose tissue within the organism.^[10]^ Thus, BMI and waist circumference have emerged as 2 pivotal metrics for evaluating obesity.^[10,11]^ Numerous observational studies have demonstrated a positive correlation between elevated BMI values and an increased predisposition towards frailty, thereby suggesting obesity as a potential hazardous determinant of frailty.^[12–15]^ Nevertheless, it is crucial to note that observational studies are prone to multiple biases and may not furnish robust causative evidence due to the intrinsic presence of reverse causation and residual confounding variables, thus limiting our comprehensive comprehension of obesity’s impact on frailty.

Mendelian randomization (MR) constitutes a sophisticated and innovative genetic epidemiological technique, utilizing genetic variants as instrumental variables (IVs) to evaluate the causality of observed associations between exposures and outcomes, effectively circumventing complications associated with confounding factors and reverse causality.^[16]^ MR is frequently extolled as a “quasi-natural experiment,” paralleling the random allotment of alleles during meiotic division to the random assignment of interventions in a traditional randomized controlled trial.^[17]^ In recently, MR investigations have garnered substantial acclaim across a variety of medical disciplines, yielding compelling, and persuasive clinical insights.^[18,19]^ To our knowledge, no antecedent study has endeavored to employ the MR framework to elucidate the causal nexus between BMI, waist circumference, and frailty risk. Therefore, the primary objective of our study is to implement a two-sample MR approach to explicate the hypothesized causality underlying the association between obesity and frailty.

## 2. Methods

### 2.1. Study design

**Figure 1.**
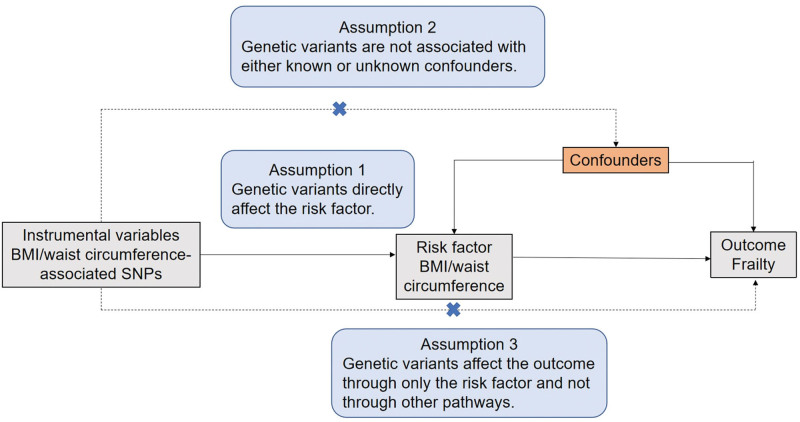
The 3 main assumptions of Mendelian randomization analysis. BMI = body mass index, SNPs = single nucleotide polymorphisms.

The MR approach is predicated on 3 fundamental assumptions (Fig. 1). Firstly, the IVs employed, comprising genetic variants, should exhibit an association with the risk factor under investigation. Secondly, these genetic variants should not demonstrate any association with confounding factors that may influence the relationship between the risk factor and the outcome of interest. Thirdly, the effects of the genetic variants on the outcome should be mediated solely through their influence on the risk factor, and not through alternative pathways.

### 2.2. Data source

Through an exhaustive exploration within the FinnGen R9 database, we obtained a dataset associated with BMI. In addition, we confined the genetic makeup of the study cohort solely to individuals of European ancestry, thus mitigating any bias that may have arisen from inter-population blending. The dataset relating to BMI (GAWS ID: finn-b-E4_HYTHY_AI_STRICT) consists of 198,472 individuals of European origin, including 22,997 cases and 175,475 healthy controls. Similarly, data on genetic variant exposure variables linked to waist circumference were derived from the IEU analysis of UK Biobank phenotypes (GAWS ID: ieu-a-61), encompassing 232,101 participants and 2,565,408 single nucleotide polymorphisms (SNPs).

The frailty data was obtained from a publicly available GWAS dataset (GWAS ID: ebi-a-GCST90020053), consisting of a cohort of 175,226 individuals of European ancestry. Janice et al acquired this dataset by conducting a GWAS meta-analysis of the Frailty Index among UK Biobank participants (n = 164,610, aged 60–70 years) and Swedish TwinGene participants (n = 10,616, aged 41–87 years). All datasets utilized in this study are publicly accessible, obviating the need for ethical approval.

### 2.3. Selection of genetic instruments

**Figure 2.**
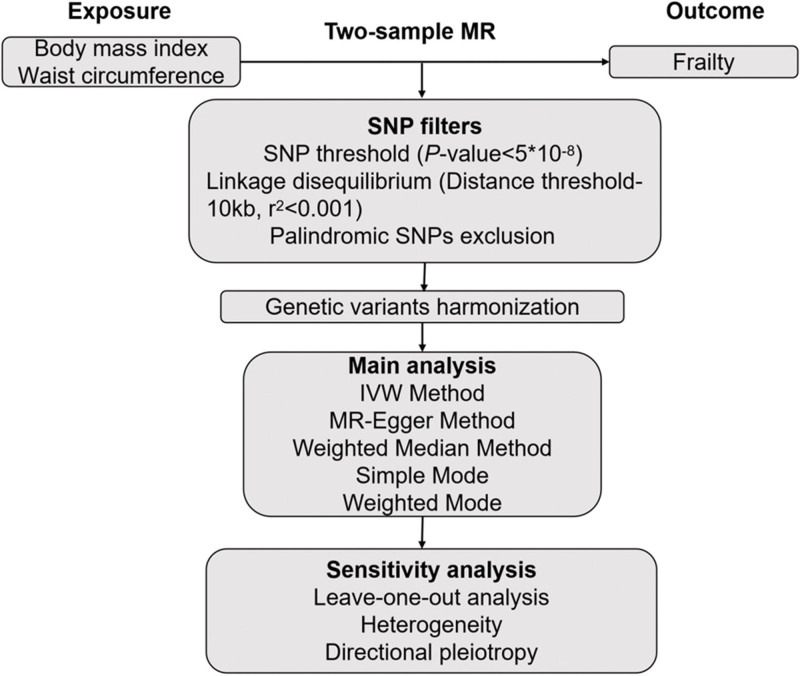
Schematic diagram of the Mendelian randomization analysis process. IVW = inverse variance weighting, MR = Mendelian randomization, SNPs = single nucleotide polymorphisms.

Corresponding genetic variants were extracted as IVs. In this phase, it is imperative to validate the SNPs as robust IVs by fulfilling the following criteria: SNPs were required to demonstrate a robust association with the exposure of interest, meeting the genome-wide significance threshold of *P* < 5.0 × 10⁻⁸; SNPs were required to be independent of one another to minimize biases arising from linkage disequilibrium, applying a threshold of *r*² < 0.001 within a 10-kilobase window, based on the European reference panel from the 1000 Genomes Project; and SNPs should ideally exert an impact on the outcome beyond their influence on the exposure. Pertinent information, including chromosome location, gene annotation, effect allele, non-effect allele, effect allele frequency, effect sizes, standard error, and *P*-value was extracted. In order to mitigate instrumental bias stemming from weak instruments, we calculated the F-statistic (s [F = *R*^2^ × (N − 2)/(1 − *R*^2^)]) for the selected SNPs to assess the strength of our IVs for the MR analysis. We evaluated the F statistic values [F=(*R*^2^/(1 − *R*^2^)) × ((N − K − 1)/K)] to assess instrument strength for the MR pairs. Briefly, N represents the sample size of the exposure data and the *R*^2^ represents the explained variance of genetic instruments. Based on beta (genetic effect size of the exposure). If the F-statistic exceeded 10, it indicated robust instrument strength. SNPs that were not present in the outcome dataset or displayed allele inconsistencies between the exposure and outcome were excluded. SNPs displaying palindromic characteristics were removed. We conducted a comprehensive search on the PhenoScanner database for all relevant SNP phenotypes and excluded those associated with potential confounders, including depression, diastolic blood pressure, heel bone mineral density, systolic blood pressure, type 2 diabetes, and overall blood pressure.^[20]^ We acquired summary statistics (beta coefficients and standard errors) for 68 and 41 SNPs associated with BMI and waist circumference as the IVs from the GWASs focusing on BMI and waist circumference respectively. A schematic representation of the step-by-step workflow implemented in this study is presented in Figure 2.

Additionally, to ensure consistency of allele definitions between the exposure and outcome datasets, we performed an allele harmonization procedure. All palindromic SNPs (i.e., A/T or C/G base pairs) were excluded. For non-palindromic SNPs, strand orientation was corrected using effect allele frequency data to ensure that the effect allele in both exposure and outcome datasets was aligned. This procedure was implemented using the harmonise_data() function in the R package TwoSampleMR.

### 2.4. Statistical analysis

The primary analysis of causal associations between exposures (BMI/waist circumference) and the outcome (frailty) was conducted using the inverse variance weighted (IVW) method. Additionally, several other approaches were employed to assess the robustness of our results, including the weighted median, MR-Egger, simple mode, and weighted mode. To account for multiple testing across the 5 MR methods, a Bonferroni correction was applied, establishing a significance threshold of *P* < .01. To examine the influence of individual SNPs on the results, we performed a leave-one-out sensitivity analysis. To further examine the robustness of results, we checked for evidence of heterogeneity (a potential indicator of pleiotropy) in IVW estimators using the Cochran *Q* statistic. We assessed heterogeneity in the IVW estimates using Cochran *Q* statistic, and when evidence of heterogeneity was detected, we applied a random-effects IVW model. The MR Egger intercept test was used to indicate the presence of directional pleiotropy. For each MR approach, we present the estimated β coefficient, its standard error, the corresponding *P*-value, and the 95% confidence interval, where applicable. The Mendelian Randomization Pleiotropy RESidual Sum and Outlier (MR-PRESSO) method was employed to detect potential outliers and, where present, to generate corrected causal estimates in the presence of horizontal pleiotropy. All statistical analyses were performed using R software (version 3.6.3; R Foundation for Statistical Computing, Vienna, Austria) with the “TwoSampleMR,” “Mendelian Randomization,” and “MR-PRESSO” packages.

## 3. Results

### 3.1. IVs for MR

**Figure 3.**
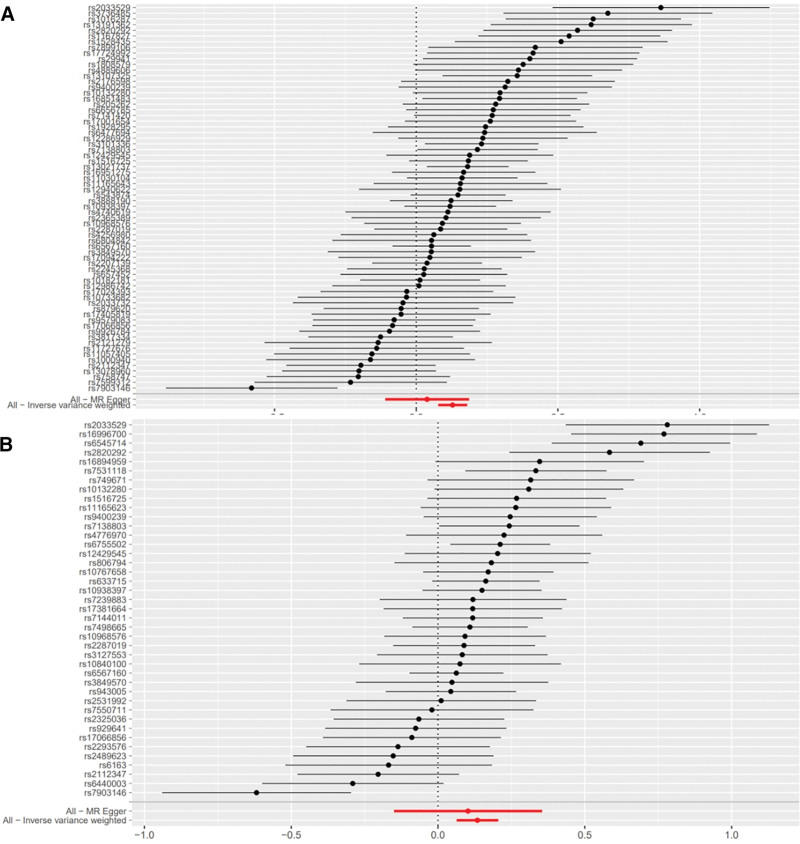
Forest plot illustrating the genetic causal relationship between BMI, waist circumference, and frailty. (A) BMI and frailty; (B) waist circumference and frailty. BMI = body mass index, MR = Mendelian randomization.

We identified 68 independent SNPs from GWASs on BMI as IVs, each exhibiting genome-wide significance for BMI (Table 1, Table S1, Supplemental Digital Content, https://links.lww.com/MD/Q964, Fig. 3A). Similarly, 41 independent SNPs were selected from GWASs on waist circumference as IVs, all meeting the criterion of genome-wide significance for waist circumference (Table 1, Table S2, Supplemental Digital Content, https://links.lww.com/MD/Q964, Fig. 3B).

**Table 1:** MR estimates from each method of assessing the causal effect of BMI and waist circumference on the risk of frailty.

MR method	Number of SNPs	Beta	SE	95% Confidence interval	*P*-value	Cochran *Q* statistic	Heterogeneity *P*‐value
*BMI*							
MR Egger	68	0.03802449	0.07504436	0.8966744 1.203353	6.140586e−01	153.8575	8.753493e−09
Weighted median	68	0.12112123	0.02675905	1.0710864 1.189543	6.000683e−06		
Inverse variance weighted	68	0.12831015	0.02557703	1.0813164 1.195353	5.258960e−07		
Simple mode	68	0.12341992	0.05969121	1.0064458 1.271776	4.253905e−02		
Weighted mode	68	0.12732230	0.03915340	1.0518827 1.226375	1.796447e−03		
*Waist circumference*							
MR Egger	41	0.1021310	0.12828161	0.8613099 1.424133	.4307662054	111.1552	7.432416e−09
Weighted median	41	0.1181818	0.03091958	1.0592694 1.195763	.0001322489		
Inverse variance weighted	41	0.1340272	0.03578463	1.0659745 1.226501	.0001801169		
Simple mode	41	0.1348853	0.05788358	1.0216648 1.281892	.0249210500		
Weighted mode	41	0.1311957	0.04835719	1.0370867 1.253545	.0097842285		

The F-statistics for the IVs associated with BMI and waist circumference all exceeded 10, while their *P*-values were consistently below 5.00E−08, indicating a robust and statistically significant association between all IVs and the respective exposures.

### 3.2. MR results

**Figure 4.**
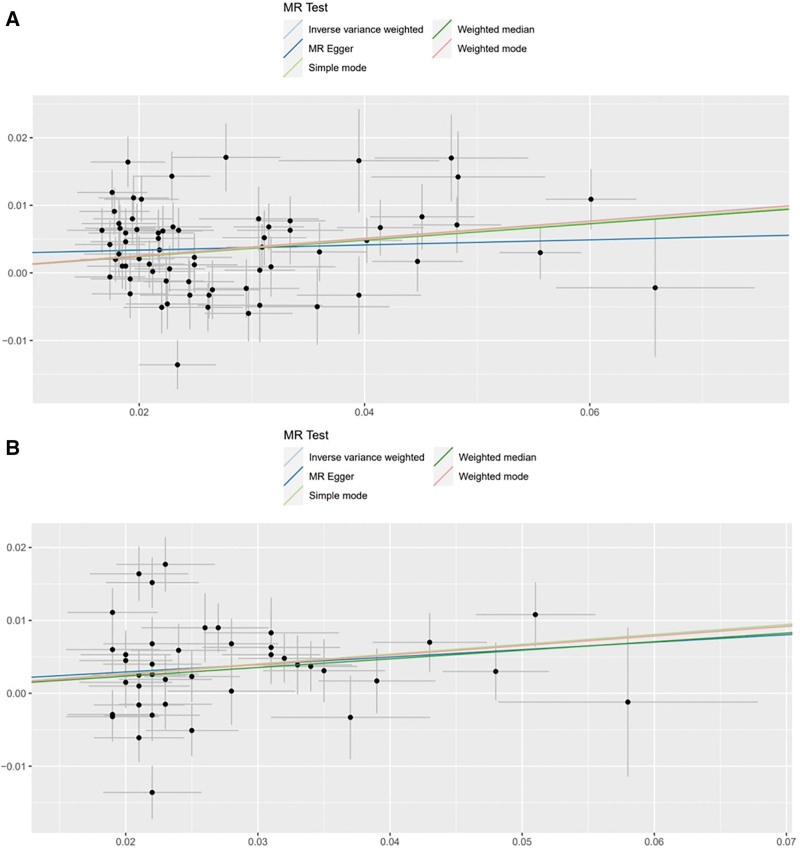
Scatter plot of genetic causality between BMI, waist circumference, and frailty using various MR methods. (A) BMI and frailty; (B) waist circumference and frailty. MR test: light blue indicates inverse-variance weighting; dark green, weighted median; dark blue, MR Egger; pink, weighted mode; and light green, simple mode. BMI = body mass index, MR = Mendelian randomization.

The IVW method showed evidence to support a causal association between BMI, waist circumference and frailty (beta = 0.12831015, SE = 0.02557703, *P* = 5.258960e−07; beta = 0.1340272, SE = 0.03578463, *P* = .0001801169), both of which remained significant after Bonferroni correction (*P* < .01). The MR-Egger analysis showed no statistically significant causal association between BMI and frailty (beta = 0.0380, SE = 0.0750, 95% CI: 0.8966–1.2033, *P* = .614) or between waist circumference and frailty (beta = 0.1021, SE = 0.1283, 95% CI: 0.8613–1.4241, *P* = .431). The wide confidence intervals overlapping zero suggest that the MR-Egger estimates are imprecise and should be interpreted with caution, particularly in the context of potential pleiotropy. However, the weighted median approach yielded evidence of a causal association between BMI, waist circumference and frailty (beta = 0.12112123, SE = 0.02675905, *P* = 6.000683e−06; beta = 0.1181818, SE = 0.03091958, *P* = .0001322489). The Simple mode approach yielded evidence of a causal association between BMI, waist circumference and frailty (beta = 0.12341992, SE = 0.05969121, *P* = 4.253905e−02; beta = 0.1348853, SE = 0.05788358, *P* = .0249210500). Besides, the weighted mode approach yielded evidence of a causal association between BMI and frailty (beta = 0.12732230, SE = 0.03915340, *P* = 1.796447e−03; beta = 0.1311957, SE = 0.04835719, *P* = .0097842285; Table 1, Figs. 3A, B and 4A, B).

The association between BMI, waist circumference and frailty were not consistent between MR Egger methods. The IVW, weighted median method, simple mode, and weighted mode suggest a causal effect of BMI and waist circumference on the risk of frailty, whereas the MR-Egger method suggests a null causal effect. Considering that compared to the MR-Egger analysis, the weighted median estimator has the advantage of retaining greater precision in the estimates, the results of the MR analysis may support a potential causal association between BMI, waist circumference and frailty.

### 3.3. Heterogeneity and sensitivity test

**Figure 5.**
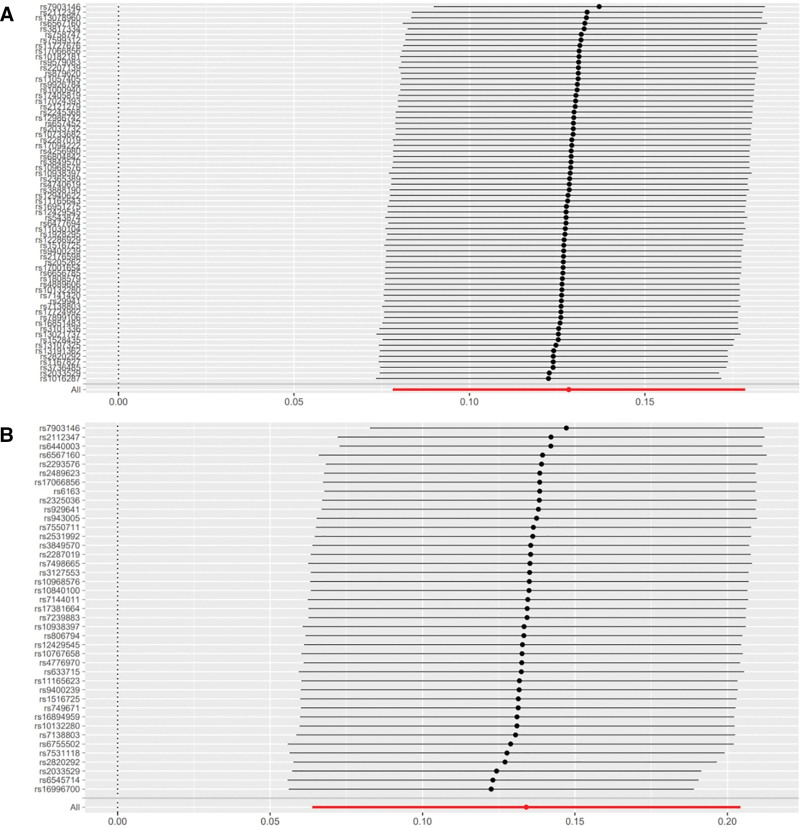
Forest plot for leave-one-out sensitivity analysis of genetic causality between BMI, waist circumference, and frailty. (A) BMI and frailty; (B) waist circumference and frailty. BMI = body mass index.

**Figure 6.**
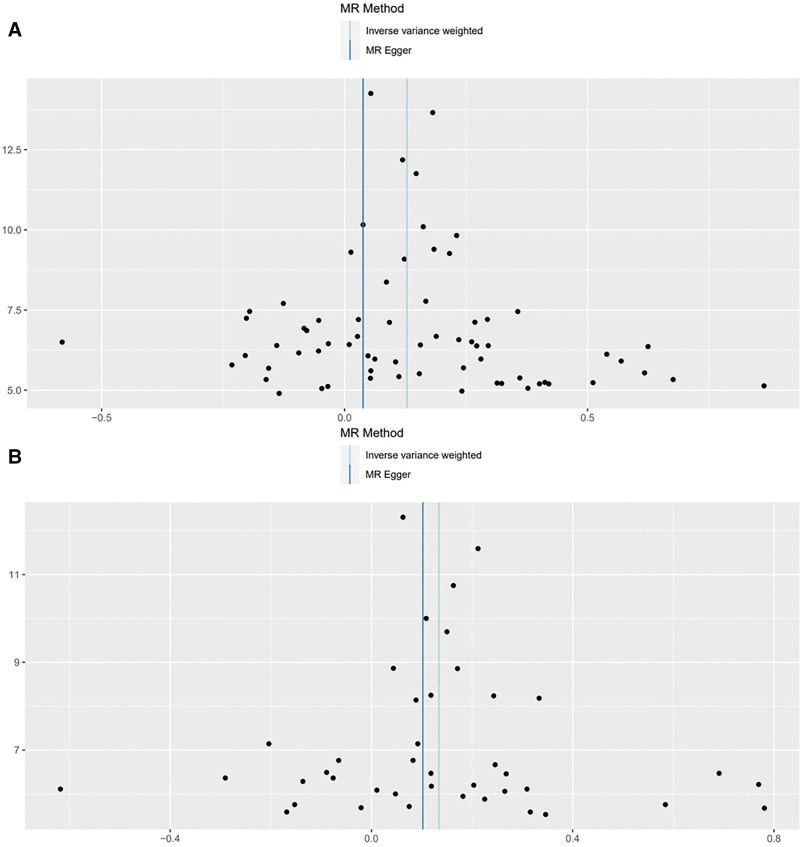
Funnel plot of genetic causality between BMI, waist circumference, and frailty. (A) BMI and frailty; (B) waist circumference and frailty. MR method: light blue indicates inverse variance weighted and dark blue, MR Egger. BMI = body mass index, MR = Mendelian randomization.

In both analyses, Cochran *Q* test revealed significant heterogeneity among the variant-specific estimates (*Q* = 153.86, *P* = 8.75 × 10⁻⁹ for BMI; *Q* = 111.16, *P* = 7.43 × 10⁻⁹ for waist circumference). Nonetheless, the concordance of causal estimates across multiple MR approaches (IVW, weighted median, simple mode, and weighted mode), together with the absence of evidence for horizontal pleiotropy based on the MR-Egger intercept, supports the robustness of our principal findings. To further address heterogeneity, we applied a random-effects IVW model, which yielded results consistent with those of the primary analysis. Besides, no significant pleiotropy was detected for the association of BMI, waist circumference and with frailty (intercept = 0.002609868, *P* = .2054007; intercept = 0.003445858; *P* = .7968586). Results from the “leave-one-out” analysis demonstrated that no single SNP was driving the IVW point estimate (Table S2, Supplemental Digital Content, https://links.lww.com/MD/Q964). As illustrated in Figure 5, the leave-one-out sensitivity analysis using a forest plot demonstrated that no single SNP exerted a disproportionate influence on the IVW point estimates. Furthermore, the funnel plots derived from both the IVW and MR-Egger analyses revealed no evidence of horizontal pleiotropy across the included studies (Fig. 6). We further applied the MR-PRESSO approach to assess and correct for potential outliers and horizontal pleiotropy. The Global Test provided no evidence of significant pleiotropy for either BMI or waist circumference in relation to frailty (*P* > .05). No outlier variants were identified, and thus no SNPs were excluded. The causal estimates remained unchanged following MR-PRESSO analysis, thereby reinforcing the robustness of our primary IVW findings.

## 4. Discussion

In this study, we explored the causal relationship between obesity and frailty using two-sample MR analyses across 3 publicly available databases. Our findings demonstrated that both BMI and waist circumference were significantly associated with an elevated risk of frailty.

To the best of our knowledge, this is the first MR study investigating the association between obesity and frailty risk through a multi-dimensional assessment of waist circumference and BMI. This underscores the significance of concurrently assessing waist circumference and BMI, as BMI alone may not suffice as the sole indicator of obesity. Specifically, in elderly individuals exhibiting waist obesity, characterized by an elevated waist circumference, there exists a potential correlation with a heightened vulnerability to frailty.^[21,22]^ Our findings provide strong evidence supporting a causal relationship between obesity and increased frailty risk. Although heterogeneity was observed, this does not inherently undermine the validity of MR analysis. Such heterogeneity may arise from variation in instrument strength, subtle differences in sample structure, or potential weak instrument bias. Nevertheless, we confirmed the robustness of the primary findings through multiple sensitivity analyses (including MR-Egger, weighted median, and leave-one-out analyses), which revealed no evidence of substantial horizontal pleiotropy or influential outliers. Accordingly, we consider that the observed heterogeneity does not diminish the central conclusion of this study – namely, that obesity exerts a positive causal effect on the risk of frailty. Although the MR-Egger intercept test did not reveal evidence of significant directional pleiotropy, the slope estimates derived from MR-Egger were nonsignificant and accompanied by broad confidence intervals, reflecting its comparatively limited statistical power relative to IVW and weighted median approaches. This limitation is well recognized, particularly when genetic instruments account for only a modest proportion of variance in the exposure. Nonetheless, the concordance of results across multiple complementary MR methods (IVW, weighted median, simple mode, and weighted mode) reinforces the robustness and credibility of our principal findings.

The complex relationship between obesity and frailty can be elucidated through a multitude of direct and indirect pathways. Primarily, adipose tissue functions as a metabolically active depot, regulating the secretion of various cytokines, such as adiponectin, interleukin-6, and tumor necrosis factor. These cytokines play crucial roles in fostering a milieu conducive to inflammation, metabolic aberrations, and the communication of metabolic signaling across diverse organ systems.^[23–25]^ As a result, this cascade instigates a detrimental sequence of events, culminating in the decline of skeletal muscle mass and strength, as corroborated by existing literature.^[26,27]^ Additionally, chronic inflammation arises as a consequence of adipose tissue infiltration, and excessive adiposity deposition within muscle fibers contributes to the impairment of muscle function. This intricate interplay between adiposity and muscle dysfunction has been labeled as “sarcopenic obesity.”^[28]^ The multifaceted phenomenon of sarcopenic obesity not only adversely affects quality of life, physical capacity, and metabolic equilibrium, but also manifests a strong correlation with increased vulnerability to disability and frailty.^[29,30]^ Moreover, it is noteworthy that elevated overall adiposity bestows a heightened predisposition towards a range of comorbidities in the elderly population, including, but not limited to, higher incidences of depression, cognitive decline, and osteoporosis.^[31,32]^ These interconnected factors are postulated as potential intermediaries along the complex pathophysiological trajectory leading to frailty.

This investigation has revealed a significant correlation between overweight and obesity status and increased frailty susceptibility, in contrast with individuals presenting a normal BMI. This observation resonates with the findings of a preceding meta-analysis, which identified an increased probability of frailty among older adults sporting a BMI ≥ 35.0 kg/m^2^.^[33]^ The conceivable mechanisms that connect adiposity to adverse outcomes primarily revolve around complex metabolic modifications and mechanical load dynamics.

Proliferation of adipocytes, particularly within the intra-abdominal fat storage, imparts traits of metabolic syndrome, encompassing insulin resistance, type 2 diabetes, hypertension, and dyslipidemia. Moreover, adipose tissue interacts intricately with inflammatory cascades, oxidative stress pathways, and immune dysregulation, which underpin the pathogenesis of cardiovascular diseases, cognitive impairment, stroke, and various malignancies.^[24,34–36]^ Additionally, excessive body weight burdens the musculoskeletal system, resulting in degenerative musculoskeletal conditions such as knee osteoarthritis, low back pain, and plantar fasciitis.^[37,38]^ Overweight and obese individuals also confront an increased vulnerability to depression.^[39]^ Importantly, frailty is not confined to individuals with decreasing or underweight statuses. Rather, the concurrent burden of obesity-related disorders and conditions significantly contributes to the accumulation of health deficits, thereby escalating the future risk of frailty.

Frailty serves as a quantifiable clinical marker, with intricate ties to adverse clinical outcomes.^[40,41]^ A recent investigation has unveiled a significant association between frailty and both mortality and length of hospital stay among patients.^[42]^ Therefore, it is essential for future research to explore the potential impact of interventions targeting BMI on frailty, and whether such interventions indirectly affect unfavorable clinical outcomes. It is salient that while the World Health Organization defines a normal BMI range as 18.5 to 24.9, discerning an appropriate BMI range to potentially attenuate frailty in elderly adults demands comprehensive consideration of individual factors and characteristics of lifestyle-related diseases.^[43]^ Notably, our meta-analysis divulges that the BMI range associated with the lowest risk of frailty in community-dwelling older adults is 18.5 to 29.9 kg/m^2^. This suggests that both overweight and normal-weight older individuals display diminished vulnerability to conditions related to frailty.

Therefore, policymakers should enhance public health initiatives, implement more rigorous weight management strategies, and prioritize the preservation of muscle mass in obese individuals within clinical practice to mitigate the risk of frailty. In recent years, advanced medical image processing approaches, including multimodal crossover global learnable attention networks, have emerged to enhance the quality of magnetic resonance imaging, offering promising avenues for the precise evaluation of obesity-related morphometric indicators.^[44]^

## 5. Conclusions

In conclusion, this MR study provides compelling evidence that increased BMI and waist circumference are significantly and positively causally associated with frailty, indicating that obesity contributes to frailty development. A more profound understanding of these relationships could significantly inform the design of targeted prevention strategies, ultimately alleviating the burden of frailty and enhancing health outcomes across diverse populations.

## Author contributions

**Writing – original draft:** Yongguang Wang, Jinlei Zhou, Tingxiao Zhao.

**Writing – review & editing:** Qi Wang.

## Supplementary Material


